# HIV comprehensive knowledge and prevalence among young adolescents in Nigeria: evidence from Akwa Ibom AIDS indicator survey, 2017

**DOI:** 10.1186/s12889-019-7890-y

**Published:** 2020-01-13

**Authors:** Titilope Badru, Jefferson Mwaisaka, Hadiza Khamofu, Chinedu Agbakwuru, Oluwasanmi Adedokun, Satish Raj Pandey, Patrick Essiet, Ezekiel James, Annie Chen-Carrington, Timothy D. Mastro, Sani H. Aliyu, Kwasi Torpey

**Affiliations:** 1 FHI 360 Nigeria, Abuja, Nigeria; 2 University of Ghana College of Health Sciences, Accra, Ghana; 3 Akwa Ibom State Ministry of Health, Uyo, Nigeria; 4 United States Agency for International Development, Abuja, Nigeria; 5 FHI 360 NC, Durham, North Carolina USA; 6 National Agency for the Control of HIV/AIDS, Abuja, Nigeria

**Keywords:** Comprehensive HIV knowledge, Stigma, Risk perceptions, Young adolescents, Nigeria

## Abstract

**Background:**

Despite the recent increase in HIV infections among adolescents, little is known about their HIV knowledge and perceptions. This study, therefore, sought to examine the factors associated with comprehensive HIV knowledge, stigma, and HIV risk perceptions among young adolescents aged 10–14 years in Akwa Ibom State, Nigeria. Additionally, consenting parents and assenting young adolescents were tested for HIV.

**Methods:**

We used cross-sectional data from the 2017 Akwa Ibom AIDS Indicator Survey to analyze comprehensive HIV knowledge, stigma, and HIV risk perceptions among young adolescents. Demographic characteristics of young adolescents were summarized using descriptive statistics. Chi-square test (or Fisher’s exact test in cases of small subgroup sample sizes) was used to elicit associations between demographics and study outcomes. Separate multivariable logistic regression models were then conducted to determine associations with the study outcomes. Sampling weights were calculated in order to adjust for the sample design. *P*-values less than 0.05 were considered to be significant.

**Results:**

A total of 1818 young adolescents were interviewed. The survey highlighted significant low levels of comprehensive HIV knowledge (9.4%) among young adolescents. Adolescent-parent discussions [AOR = 2.19, 95% C.I (1.10–4.38), *p* = 0.03], schools as sources of HIV information [AOR = 8.06, 95% C.I (1.70–38.33), *p* < 0.001], and sexual activeness [AOR = 2.55, 95% C.I (1.16–5.60), *p* = 0.02] were associated with comprehensive HIV knowledge. Majority (93%) of young adolescents perceived themselves not to be at risk of HIV. Overall, 81.5% of young adolescents reported stigmatizing tendencies towards people living with HIV. HIV prevalence among young adolescents was 0.6%.

**Conclusions:**

Results indicate low comprehensive HIV knowledge among young adolescents. Our findings suggest that there is a need for increased attention towards young adolescents particularly in the provision of comprehensive, functional sexuality education, including HIV at the family- and school-levels. Consequently, age appropriate interventions are needed to address the epidemiological risks of young adolescents that are influenced by a myriad of social issues.

## Background

Young people today have more sources of information for improving their HIV knowledge such as family members, friends, teachers, and the Internet. Adequate HIV knowledge is critical for protecting young adolescents from HIV as evidence has shown that they are among the most vulnerable groups [1]. In spite of these many sources, HIV prevalence among young adolescents remains a public health concern. In 2015, it was estimated that globally 29 adolescents acquired HIV every hour and that approximately 1.8 million adolescents aged 10–19 years were living with HIV, majority of whom were girls [2]. HIV deaths among adolescents in Africa continue to rise at an alarming rate [3]. AIDS is currently the number one cause of death among adolescents in Africa, and second leading cause of adolescent deaths worldwide, with sub-Saharan Africa having the highest number of deaths [4]. The number of adolescents dying from HIV related illnesses is estimated to have tripled over the last two decades [4]. An estimated 1.9 million people are living with HIV in Nigeria accounting for a prevalence of 1.4%. Among children aged 0–14 years, HIV prevalence is estimated to be 0.2% [5]. The HIV prevalence of adolescents in Nigeria is estimated to be 3.5%, the highest among countries in West and Central Africa [6]. UNICEF in 2017 estimated in Nigeria 230,000 adolescents aged 10–19 live with HIV and 5400 have succumbed to AIDS-related deaths [3]. As adolescents and young people continue to be disproportionately affected by HIV, global and national efforts should focus on shifting the age disaggregation to accommodate young adolescents aged 10–14, as they tend to be overlooked by interventions due to programs prioritizing 15–19-year-old adolescents and young people aged 20–24. The 2014 Nigeria Demographic and Health Survey (NDHS) reported that 89.3% of boys and 89.5% of girls aged 15–19 had heard of AIDS. On HIV prevention methods, 63% of boys compared to 51.6% of girls knew that consistency in condom use could reduce the risk of HIV infection [7]. Young adolescents aged 10–14 were not included in the NDHS, except for when adults 18–49 responded on whether children aged 12–14 should be provided knowledge on condom use for HIV prevention. Lack of data for young adolescents aged 10–14 makes it difficult for them to be included in the national strategic plans, thereby limiting the available evidence to inform age-specific programming that targets young adolescents [8].

There has also been a decline in formal sex education given to young adolescents, specifically on topics discussing abstinence, birth control, and prevention of HIV/AIDS and other STDs [9]. This has mainly been reported in the Western countries, whereas Nigeria remains in limbo as to whether such topics should be discussed in the open or not. As a result, young people in Nigeria, especially young adolescents aged 10–14, face substantial challenges in accessing timely and appropriate health education, including comprehensive sexuality education [10]. This may be attributed to societal attitudes and misperceptions about sexuality education, therefore exposing young adolescents to other unreliable sources of information. Parent-child sexual communication plays a protective role in adolescent safer sex behaviors, including condom use [11]. In Nigeria, age, religion, and socioeconomic status have been found to be positive influencers for parent-child communication [12]. In addition, most parents tend to communicate sexual matters to their children after they have already engaged in sexual acts [12] .

Inadequate HIV knowledge among young adolescents coupled with socio-cultural factors may contribute to stigmatizing tendencies towards those infected and affected by HIV. If not addressed, increased stigma and discrimination, especially against young adolescents will continue to hinder them from testing and adhering to treatment. Among the general population in Nigeria, the HIV stigma level has declined [13], however this is yet to be determined among young adolescents aged 10–14. Similarly on risk perceptions, some studies in Africa [14–16] have found perceived low and inaccurate reporting of HIV risks among adolescents. It is therefore beneficial to accurately understand why young adolescents perceive themselves to be at low risk in order to address misconceptions associated with their beliefs. Understanding and addressing HIV knowledge gaps among young adolescents is therefore critical for programs and policy makers when designing behavior change interventions. This paper, therefore, sought to determine the factors associated with comprehensive HIV knowledge, HIV perceptions, stigma, and sexual behaviors among young adolescents in Akwa Ibom State Nigeria.

## Methods

### Survey methodology

The Akwa Ibom AIDS Indicator Survey (AKAIS) was conducted between April and June 2017 among children aged 0 months to 14 years and adults 15 years and older. AKAIS was a population-based survey of household residents designed to produce unbiased estimates of HIV prevalence and incidence, and to identify the risk factors associated with HIV infection in Akwa Ibom state. It was estimated that a sample of 4653 households within 226 Enumeration Areas (EAs) would provide a representative sample of adults aged 15 years and older and children aged 0 months to 14 years.

A two-stage probability sampling technique was employed in selecting participants from a frame of eligible household residents of Akwa Ibom State. The primary sampling unit was EAs as defined by the National Population Commission (NPC) during the 2006 Nigeria Census. At the first stage, 226 clusters (EAs) were selected with probability proportional to size and stratified by geographic location. At the second stage, a fixed number of households within the selected EAs were selected using systematic sampling. A complete listing of all households in selected EAs was conducted. All adults 15 years and older and young adolescents 10–14 years in the sampled households, who were either permanent residents or visitors in the household on the night preceding the survey, were eligible for the interview and/or HIV testing. Similarly, all children less than 10 years were eligible for HIV testing.

Tablet-based questionnaires used for this study was adapted from the AIDS Indicator Survey tool (Additional file 1, Akwa Ibom AIDS Indicator Survey Adolescent Individual Questionnaire [10–14 yrs.]) were administered through face-to-face interviews. Three types of questionnaires were used: (1) a household questionnaire, (2) an individual adolescent questionnaire for individuals aged 10–14 years, and (3) an individual adult questionnaire for women and men aged 15 years or older. The individual adolescent and adult questionnaires collected information from eligible adolescents aged 10–14 years and adults aged 15 years and older on basic demographic characteristics, marriage, sexual activity, HIV and STI knowledge, attitudes and behaviors, and previous HIV testing. In addition to the interview, blood was drawn from consenting participants for HIV antibody testing. Informed consent was sought for participation in the interview and blood draw. Parental consent was sought from the parent or guardian of children less than 17 years. In addition, assent was sought from children aged 10–17 years whose parent or guardian had consented to their participation. Consenting participants were tested for HIV according to national algorithm and confirmed with Bio-Rad Geenius HIV 1/2 Confirmatory Assay. Personal identifiers were excluded from the data set before analyses were performed.

### Study measures

The adolescent questionnaire elicited information on demographic characteristics, comprehensive knowledge of HIV, attitudes, HIV risk perception, HIV testing, and alcohol and drug use. Adolescents aged 12–14 years were additionally asked questions about sexual activity, social norms, abstinence, self-efficacy, and assertiveness.

In this study, we analyzed the following outcomes reported by adolescents aged 10–14 years: comprehensive HIV knowledge, stigma, and HIV risk perceptions. HIV/AIDS awareness was assessed by asking adolescents if they had ever heard of HIV/AIDS. Comprehensive knowledge of HIV was assessed, and this was defined as: i) knowing that someone can protect himself/herself from HIV by using condom during sexual intercourse, ii) knowing that a healthy-looking person can have HIV, iii) knowing that HIV can be transmitted by having unprotected sex with an HIV-infected person, iv) knowing that there are medicines that people with HIV can take to help them live longer, and v) knowing that HIV can be transmitted by sharing of sharp objects. A binary outcome of “1” was designated if all questions were answered correctly and “0” if any of the questions were answered incorrectly.

Stigma was assessed by asking the following questions: i) would you be willing to share food with an HIV-infected person? and ii) would you play with someone who has HIV? For the stigma-related outcome, these questions were combined. HIV risk perception was assessed by asking adolescents the following question: “How likely do you think is it that you can get HIV: Very Likely, Somewhat Likely, or Not Likely? A binary outcome of “1” was designated if adolescents reported very likely or somewhat likely and “0″ if adolescents reported not likely. Independent variables included: sex, educational status, location/residence, ever had sex, having discussed HIV with parents/guardians, and ever tested for HIV.

### Data analysis

Adolescent characteristics including age, gender, and level of education were summarized using descriptive statistics. Ever heard of HIV, ever had sex, condom use, and having HIV discussions with parents/guardians were reported using weighted proportions and 95% confidence intervals. Chi-square test (or Fisher’s exact test in cases of small subgroup sample sizes) was used to elicit associations between demographics and HIV/AIDS awareness and ever had sex. Separate multivariable logistic regression models were conducted to determine associations with comprehensive HIV knowledge, HIV risk perception, and stigma. Sampling weights were calculated in order to adjust for the sample design. *P*-values less than 0.05 were considered to be significant. Statistical analyses were performed using Stata 12.0 (StataCorp, 2012, Stata Statistical Software: Release 12.0, College Station, TX: StataCorp LP).

## Results

### Characteristics of the respondents

A total of 2076 adolescents (10–14 years) were eligible for the survey, 1818 participated in the interviews. Interview response rate amongst adolescents was 87.6%. Of the 1818 adolescents interviewed, 70% (1281) resided in rural areas and 53% (972) were males. The mean age was 11.9 ± 1.4 years. Majority (97%, or 1770) were currently in school, 96% (1765) had at least a primary education. Almost two-thirds (64%) were aged 10–12 years and none of the adolescents reported ever being married. Among the adolescents surveyed, 732 (40.4%) reported ever drinking alcohol and 25 (1.4%) reported ever taking mood-enhancing drugs/substances.

### HIV/AIDS awareness

Approximately 72% (1286) of young adolescents reported to have heard of HIV. Awareness of HIV or AIDS was higher among adolescents who resided in urban areas (79.7%) than rural areas (68.1%) (*p* < 0.001). HIV awareness did not differ by sex (females 73.1% vs males 70.6%; *p* = 0.32) (Table 1).

**Table 1:** HIV/AIDS Awareness amongst young adolescents by select characteristics

Select Characteristics	Awareness of HIV/AIDS among adolescents		
	Unweighted	Weighted %	*p*-value
Sex			
Male	972	70.6	0.32
Female	846	73.1	
Location			
Urban	537	79.7	< 0.001
Rural	1281	68.1	
Education			
No education	53	61.0	0.09
At least primary education	1765	72.0	

Major sources of HIV information were schools (79.7%), media (31.9%), and friends (20.9%). Other sources of information included religious leaders (7.0%), hospitals (3.6%), and the Internet (1.5%). Slightly over a quarter (26.2%) of adolescents ever discussed HIV or AIDS with their parents or guardians (males 26.1% vs females 25.3%, *p* = 0.64). Almost three out of ten adolescents who resided in the rural areas (28.5%) and 21.6% who resided in urban areas had ever discussed HIV or AIDS with their parents or guardians (*p* = 0.02).

### HIV/AIDS knowledge

**Fig. 1:**
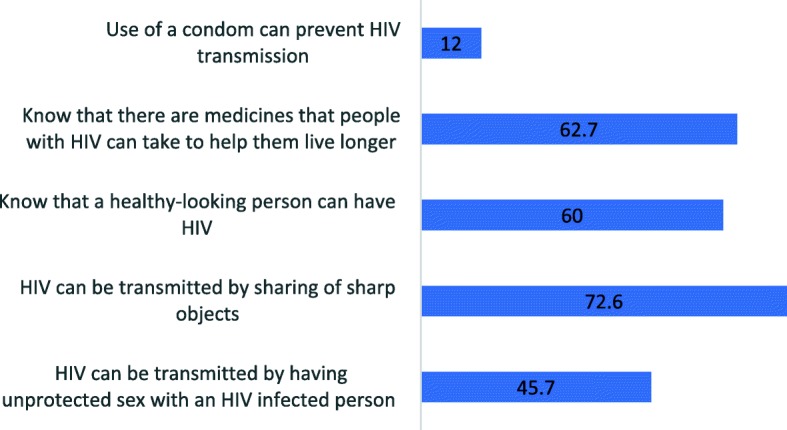
Percentage of adolescents who answered the HIV knowledge questions correctly. Method and results: Proportion of young adolescents who had ever heard of HIV and gave correct responses on HIV knowledge questions including mode of HIV transmission, HIV misperceptions, HIV treatment and HIV prevention. Data is shown as a percentage of 1286 adolescents who had ever heard of HIV

Out of the 1286 adolescents who had ever heard of HIV, almost half (45.7%) of the adolescents answered correctly that HIV can be transmitted by having unprotected sex with an HIV-infected person and by sharing sharp objects (72.6%). Sixty percent of the adolescents knew that a healthy-looking person can have HIV and 62.7% knew that there are medicines that people with HIV can take to help them live longer. Only 12% knew that the use of a condom can prevent HIV transmission (Fig. 1).

Almost one out of ten (9.4%) adolescents had comprehensive knowledge of HIV. A multivariable logistic regression of factors associated with HIV comprehensive knowledge indicated that adolescents who had ever had a discussion on HIV or AIDS with their parents/guardians [AOR = 2.19, 95% C.I (1.10–4.38), *p* = 0.03] were more likely to have comprehensive knowledge of HIV than those who had never had a discussion on HIV or AIDS with their parents/guardians. Adolescents who had ever had sex were more likely [AOR = 2.55, 95% C.I (1.16–5.60), *p* = 0.02] to have comprehensive knowledge of HIV than adolescents who had never had sex. Adolescents whose source of HIV information was from schools [AOR = 8.06, 95% C.I (1.70–38.33), *p* < 0.001] were more likely to have comprehensive knowledge of HIV than those who did not receive HIV information from schools (Table 2).

**Table 2:** Factors associated with HIV comprehensive knowledge among adolescents

	Crude OR (95% C.I)	*P*-value	Adjusted Crude OR (95% C.I)	*P*-value
Location				
Urban	1		1	
Rural	0.91 (0.61–1.37)	0.66	0.91 (0.44–1.89)	0.80
Sex				
Male	1		1	
Female	1.06 (0.72–1.54)	0.78	0.75 (0.37–1.49)	0.41
Age (years)	1.41 (1.22–1.65)	< 0.001	1.45 (0.96–2.20)	0.08
Education				
No form of education	1		1	
At least primary education	1.02 (0.35–2.93)	0.98	0.35 (0.09–1.44)	0.15
Ever discussed HIV with parents/guardians				
No	1		1	
Yes	1.89 (1.26–2.82)	< 0.001	2.19 (1.10–4.38)	0.03
Ever had sex				
No	1		1	
Yes	2.18 (1.09–4.39)	0.03	2.55 (1.16–5.60)	0.02
Ever tested for HIV				
Yes	1		1	
No	0.59 (0.32–1.09)	0.09	0.56 (0.24–1.30)	0.17
Source of HIV information: internet				
No	1		1	
Yes	5.27 (1.82–15.28)	< 0.001	0.58 (0.08–3.95)	0.58
Source of HIV information: media				
No	1		1	
Yes	2.02 (1.35–3.01)	< 0.001	1.45 (0.69–3.07)	0.33
Source of HIV information: school				
No	1		1	
Yes	11.51 (5.45–24.29)	< 0.001	8.06 (1.70–38.33)	< 0.001

### Sexual behaviors

Data on sexual behaviors were collected among respondents aged 12–14 years (1029). Forty-two percent (392) reported to have ever heard of sex while 22.1% (86) reported to have ever had sex. Of the 86 who reported to have ever had sex, 40% reported their sexual debut before the age of 12 years and 9.3% reported using condoms at first sex. Twelve percent of the sexually active respondents reported to have had sex for material support. Adolescents aged 12–14 years who had ever had sex did not differ between females (23.4%) and males (20.6%) (*p* = 0.64). Exposure to sexual intercourse did not differ by location of residence (rural 24.3% vs. urban 17.4%; *p* = 0.12) (Table 3).

**Table 3:** Ever had sex by select characteristics among adolescents aged 12–14 years

	Unweighted	Weighted %	*p*-value
Overall	392	22.1	
Sex			
Male	191	20.6	0.64
Female	201	23.4	
Location			
Urban	123	17.4	0.12
Rural	269	24.3	
Education			
No education	21	41.9	
At least primary education	371	21.0	0.09
Age group (years)			
12	114	17.8	0.17
13	121	20.2	
14	157	26.6	

### HIV risk perception

Only 7% of the adolescents aged 12–14 years who had heard of HIV perceived themselves to be at risk of HIV. A multivariable logistic regression of factors associated with HIV risk perception showed that sex, location of the respondents, ever discussed HIV with parents/guardians, ever tested for HIV, and having comprehensive knowledge of HIV were not significantly associated with HIV risk perception. Adolescents aged 12–14 years who had ever had sex were more likely to perceive themselves to be at risk of HIV [AOR = 4.18, 95% C.I (1.63–10.69), *p* < 0.001) than those who had never had sex. HIV risk perception increased with an increase in age [AOR = 1.80, 95% C.I (1.02–3.50), *p* = 0.04] (Table 4).

**Table 4:** Factors associated with HIV risk perception among adolescents

	Crude OR (95% C.I)	*P*-value	Adjusted Crude OR (95% C.I)	*P*-value
Location				
Urban	1		1	
Rural	0.94 (0.51–1.73)	0.84	0.65 (0.22–1.89)	0.43
Sex				
Male	1		1	
Female	0.91 (0.51–1.62)	0.75	0.46 (0.16–1.33)	0.15
Age (years)	1.04 (0.75–1.46)	0.80	1.80 (1.02–3.50)	0.04
Ever discussed HIV with parents/guardians				
No	1		1	
Yes	1.79 (0.99–3.24)	0.05	0.91 (0.31–2.72)	0.87
Ever had sex				
No	1		1	
Yes	3.95 (1.55–10.07)	< 0.001	4.18 (1.63–10.69)	< 0.001
Comprehensive HIV knowledge				
No	1		1	
Yes	1.45 (0.65–3.23)	0.37	0.92 (0.26–3.22)	0.89
Ever tested for HIV				
Yes	1		1	
No	1.07 (0.44–2.60)	0.89	0.81 (0.23–2.93)	0.75

### HIV stigma

Seventy-seven percent of young adolescents reported that they will be unwilling to play with someone who has HIV and 84% indicated that they will be unwilling to share food with someone who has HIV. Overall, 81.5% of adolescents reported stigmatizing attitudes towards people living with HIV. Multivariable logistic regression results revealed that adolescents without comprehensive HIV knowledge [AOR = 3.39, 95% C.I (1.57–7.31), p < 0.001] were more likely to have stigmatizing attitudes towards PLHIV than those with HIV comprehensive knowledge. Adolescents who perceive themselves not at risk for HIV [AOR = 3.07, 95% C.I (1.14–8.23), *p* = 0.03] were more likely to have stigmatizing attitudes towards PLHIV than those who perceived themselves to be at risk. Also, adolescents who reported to have never tested for HIV [AOR = 2.23, 95% C.I (1.15–4.32), *p* = 0.02] were more likely to have stigmatizing attitudes towards PLHIV than those who had ever tested for HIV. Adolescents without any form of education [AOR = 5.02, 95% C.I (1.34–18.76), p = 0.02] were more likely to have stigmatizing attitudes than those who had at least primary education (Table 5).

**Table 5:** Factors associated with HIV stigmatizing attitude among adolescents

	Crude OR (95% C.I)	*P*-value	Adjusted Crude OR (95% C.I)	*P*-value
Location				
Urban	1		1	
Rural	1.58 (1.23–2.03)	< 0.001	1.28 (0.77–2.11)	0.34
Sex				
Male	1		1	
Female	0.69 (0.54–0.88)	< 0.001	0.82 (0.51–1.33)	0.43
Age (years)	0.74 (0.68–0.81)	< 0.001	0.93 (0.69–1.24)	0.61
Education				
At least primary education	1		1	
No form of education	1.46 (0.65–3.32)	0.36	5.02 (1.34–18.76)	0.02
Ever discussed HIV with parents/guardians				
No	1		1	
Yes	0.86 (0.65–1.15)	0.31	0.93 (0.69–1.24)	0.61
Ever had sex				
No	1		1	
Yes	1.47 (0.85–2.57)	0.17	1.83 (0.94–3.55)	0.08
Comprehensive HIV knowledge				
Yes	1		1	
No	5.09 (3.45–7.52)	< 0.001	3.39 (1.57–7.31)	< 0.001
Ever tested for HIV				
Yes	1		1	
No	2.53 (1.62–3.94)	< 0.001	2.23 (1.15–4.32)	0.02
Perceived risk of HIV				
Yes	1		1	
No	2.20 (1.24–3.91)	< 0.001	3.07 (1.14–8.23)	0.03

### HIV prevalence

Majority (97%, or 1765) of the adolescents tested for HIV during the survey. Of these, 11 (0.6%) adolescents tested HIV positive. HIV prevalence was higher among adolescents who resided in rural areas than urban areas (0.9% vs. 0.0%, *p* = 0.04). HIV prevalence did not differ between male (0.8%) and female (0.4%) adolescents (*p* = 0.49) (Table 6).

**Table 6:** HIV Prevalence among adolescents by select characteristics

Select Characteristics	HIV prevalence among adolescents tested for HIV		
	Unweighted	Weighted %	*p*-value
Sex			
Male	940	0.8	0.49
Female	825	0.4	
Location			
Urban	524	0.0	0.04
Rural	1241	0.9	
Education			
No education	51	0.0	0.72
At least primary education	1714	0.6	

## Discussions

Findings from this survey established that comprehensive HIV knowledge among young adolescents is abysmally low. Education was shown to be a factor associated with HIV awareness as adolescents with at least a primary education reported high levels of HIV awareness compared to those without any formal schooling. Although this survey didn’t confirm the type of information young adolescents received from the listed sources of information, including teachers; survey findings disclosed that young adolescents who reported schools as their source of HIV information had a bigger likelihood of having comprehensive HIV knowledge compared to those who reported other sources. Similar findings were observed in young adolescents in South Africa and El Salvador where schools were the main source of sexual and reproductive health information, including HIV [17, 18]. Moreover, a viewpoint on Family Life and HIV Education (FLHE) education curriculum in Nigerian secondary schools reported higher knowledge scores on health issues related to adolescents sexuality and reproduction including HIV [19]. These findings reinforce the need for school curriculum changes that will make FLHE a compulsory subject for all students in primary and secondary schools. It has been argued that schools provide better avenues for structured and age appropriate HIV information compared to other sources [20], however, parents and caregivers are equally influential in the health and social well-being of their children. This survey found a significant association between the comprehensive HIV knowledge of respondents and parent-child HIV discussions; young adolescents who reported to have had discussions on HIV with their parents were more likely to have comprehensive HIV knowledge compared to their peers who did not have such discussions. These results relate to a study on the HIV comprehensive knowledge of young people in Western Ethiopia, which established that respondents who discussed sexual matters with their parents were 2.36 times more likely to have comprehensive HIV knowledge compared to their peers [21].

Adolescent sexual activeness was also associated with comprehensive HIV knowledge in that those who reported to be sexually active (ever had sex) were more likely to have comprehensive HIV knowledge compared to those who reported to have never had sex. Young adolescents reporting sufficient comprehensive knowledge have been evinced to be willing to engage in risky sexual behaviors with familiar people [22], as this survey recognized troubling low condom use at first sex. A similar finding was also reported in Kenya [23], where 12–14 year old adolescents reported nil condom use at first sex; very young adolescents were reported to be less likely to use a condom compared to older adolescents. This puts adolescents at greater risk of being infected with HIV. Young adolescents, therefore, need to be empowered to not only abstain from sex but build their self-efficacy to negotiate for condom use.

A sexual debut of between 12 and 14 years and the low comprehensive HIV knowledge in young adolescents validates the need to increase focus on all adolescents regardless of their age when providing comprehensive sexuality education, including HIV knowledge from as low as age 10. Targeting young adolescents with specific interventions aimed at addressing their HIV comprehensive knowledge gaps need to be the focus of the public health community; as this study established a very low proportion of young adolescents, 12% who knew that the use of a condom can prevent HIV transmission. Additionally, less than half (45.7%) of the adolescents answered correctly that HIV can be transmitted by having unprotected sex with an HIV-infected person. These findings relate to other studies in Nigeria and other African states [20, 21, 24] regarding low comprehensive HIV knowledge among adolescents. Therefore, young adolescents need early protection through repeated exposures to information and interventions, and policy makers and educators need to consider reaching out to preadolescent groups with HIV prevention and risk reduction programs as a fixed module within the education sector [25]. In addition, the transition phase from young adolescence to adulthood is mainly regarded as an experimentation phase where young people may want independence as they seek social separation from adults, including parents and other family members. However, as their brain matures, young adolescents are in a better stage to grasp key messages related to their health and social wellbeing if relevant comprehensive information is offered to them [26].

The continuous perception of fear by adults to discuss sex with young adolescents predisposes them to risky behaviors caused by ignorance fueled by taboo notions. Moreover, young adolescents consider themselves to be a low-risk population for HIV infections. The gaps in knowledge and low risk perceptions put young adolescents in a precarious state which exposes them to HIV through risky sexual behaviors influenced by optimism bias. As this survey established, the HIV prevalence among young adolescents (0.6%) was greater than that reported (0.2%) in the recently released national HIV survey results, more so when young adolescents were combined with children aged 0–9 years [5], thus missing the exact estimates. Similarly, Oginni and co-authors in trying to establish the trends and determinants of comprehensive HIV knowledge among adolescents missed out on the prevalence of young adolescents [24]. Therefore, this survey presents the first ever HIV prevalence of young adolescents in Nigeria that can be incorporated in the national HIV strategy for adolescents and young people, which reported missing estimates of HIV prevalence for young adolescents 10–14 years despite them forming the largest proportion (12.3%) of adolescents and young people in Nigeria [27].

Young adolescents with adequate HIV knowledge will most likely know how to protect themselves and are less likely to stigmatize those infected or affected as the survey observed that stigmatizing tendencies were low among young adolescents with comprehensive HIV knowledge. Comprehensive sexuality and HIV knowledge is therefore an important determinant of positive health outcomes among young adolescents; hence, the need for provision and implementation of age appropriate Comprehensive Sexuality Education (CSE) to young adolescents cannot be overemphasized. CSE should be integrated within school-, family-, and community-levels, emphasis needs to be on age-appropriateness and should begin early in life in order to empower young adolescents to take charge of their own health [28]. Likewise, relevant and age-appropriate social determinants of health need to be integrated within the health interventions targeting young adolescents as most adolescent-related factors lie outside the health system. This is yet to happen as young adolescents have consistently been invisible in many social and health related studies, surveys, and programs, resulting in their lack of consideration during policy making [29]. Appropriate and consistent age definitions coupled with sustainable information systems will make young adolescents visible to policy makers, researchers, donors, and other relevant partners while appreciating the dynamic nature of health across this young generation [30]. Involving young adolescents from designing to the actual implementation of HIV and other health related interventions should be considered by programs and policy makers. Young adolescents are not only passive recipients of HIV information and interventions but can be made effective advocates to reach out to their peers with accurate health information that would ultimately dispel inaccurate HIV-related attitudes and sexuality information provided to them.

## Study limitations

Responses were self-reported; social desirability bias from young adolescents might have led to under or over reporting, however, the representativeness of the survey sample and comparability with other studies strengthen the results. Another limitation was that, sexual behaviors’ and risk perceptions’ data for young adolescents aged 10 and 11 years was not collected. Additionally, the survey questionnaire did not capture questions that would identify adolescents with perinatal HIV infection, these specific results should therefore be interpreted with caution.

## Conclusion

The gap in comprehensive HIV knowledge, early sexual debut, and the recent increase in HIV infections among young adolescents necessitates the need for increased attention towards this age group. Preventive measures through increased comprehensive functional HIV knowledge need to be emphasized by all players in the fight against HIV infections among young adolescents. In addition, focus should not only be towards older adolescents and young people aged 15–24 but efforts should be pooled towards designing age appropriate, preventive, educational and cultural programs and interventions to reach the growing number of young adolescents in Africa with relevant sexual health information and interventions.

## Supplementary information

**Additional file 1.** Akwa Ibom AIDS Indicator Survey Adolescent Individual Questionnaire (10–14 yrs).

## Data Availability

The datasets used and analyzed in this study are available from the corresponding author upon request.

## Abbreviations

AKAIS: Akwa Ibom AIDS Indicator Survey
CSE: Comprehensive Sexuality Education
EA: Enumeration Area
NDHS: Nigeria Demographic and Health Survey

## References

[1] Agyemang S, Buor D, Tagoe-darko E (2012). The extent of knowledge about HIV / AIDS among young people in the Ejura-Sekyedumase district of Ghana. J AIDS HIV Res.

[2] UNAIDS. Ending the AIDS epidemic for adolescents, with adolescents [Internet]. Geneva; 2016. Available from: http://www.unaids.org/en/resources/documents/2016/ending-AIDS-epidemic-adolescents. Accessed 8 Mar 2019.

[3] WHO. Health for the World’s Adolescents: A second chance in the second decade [Internet]. Who/Fwc/Mca/14.05. Geneva; 2014. Available from: www.who.int/adolescent/second-decade. Accessed 8 Mar 2019.

[4] UNICEF. Adolescent deaths from AIDS tripled since 2000 [Internet]. Press release. 2015 [cited 2019 Feb 9]. Available from: https://www.unicef.org/media/media_86384.html

[5] UNAIDS Federal Ministry of Health. New survey results indicate that Nigeria has an HIV prevalence of 1.4% [Internet]. Abuja/Geneva; 2019. Available from: http://www.unaids.org/en/resources/presscentre/pressreleaseandstatementarchive/2019/march/20190314_nigeria. Accessed 8 Mar 2019.

[6] Pharr JR, Enejoh V, Mavegam BO, Olutola A, Karick H, Ezeanolue EE (2017). A Cross-Sectional Study of the Role of HIV / AIDS Knowledge in Risky Sexual Behaviors of Adolescents in Nigeria. Int J High Risk Behav Addict.

[7] National Population Commission, ICF International. Nigeria Demographic and Health Survey [Internet]. Abuja; 2014. Available from: https://dhsprogram.com/pubs/pdf/fr293/fr293.pdf. Accessed 8 Mar 2019.

[8] STOPAIDS. Adolescents and young people and HIV [Internet]. 2016. Available from: https://stopaids.org.uk/wp/wp-content/uploads/2017/06/STOPAIDS-Factsheet-Adolescents-and-young-people-and-HIV.pdf. Accessed 8 Mar 2019.

[9] Lindberg LD, Maddow-zimet I, Boonstra H (2016). Changes in Adolescents ’ Receipt of Sex Education , 2006-2013. J Adolesc Heal.

[10] UNAIDS. Active involvement of young people is key to ending the AIDS epidemic by 2030 [Internet]. Update. 2015 [cited 2019 Feb 9]. Available from: http://www.unaids.org/en/resources/presscentre/featurestories/2015/august/20150812_PACT

[11] Widman L, Choukas-bradley S, Noar S, Helms SW, Nesi J, Garret K (2016). Parent-Adolescent Sexual Communication and Adolescent Safer SEx Behaviour: A Meta-Analysis. JAMA Pediatr.

[12] Berg K, Sun CJ, Babalola S (2012). Predictors of parent – child communication among a nationally representative sample in Nigeria. J Soc Asp HIV/AIDS.

[13] Odimegwu CO, Akinyemi JO, Alabi OO. HIV-Stigma in Nigeria : Review of Research Studies , Policies , and Programmes. AIDS Res Treat. 2017;2017. Available from: https://www.hindawi.com/journals/art/2017/5812650/. Accessed 8 Mar 2019. 10.1155/2017/5812650 PMC5763061 29445545

[14] Price JT, Rosenberg NE, Vansia D, Phanga T, Bhushan NL, Maseko B (2018). Predictors of HIV , HIV Risk Perception , and HIV Worry Among Adolescent Girls and Young Women in Lilongwe, Malawi. J Acquir Immune Defic Syndr.

[15] Maughan-brown B, Venkataramani AS (2018). Accuracy and determinants of perceived HIV risk among young women in South Africa. BMC Public Health.

[16] Chinonyelum K, Onyechi N, Eseadi C, Okere AU, Otu MS. Effects of Rational-Emotive Health Education Program on HIV risk perceptions among in-school adolescents in Nigeria. Medicine (Baltimore). 2016;95(29) Available from: https://www.ncbi.nlm.nih.gov/pmc/articles/PMC5265750/pdf/medi-95-e3967.pdf. Accessed 8 Mar 2019. 10.1097/MD.0000000000003967 PMC5265750 27442633

[17] Cortez R, Revuelta K-A, Guirola Y, Gordillo-Tobar A. Adolescent Sexual and Reproductive Health and Rights in El Salvador [Internet]. 2016. Available from: https://www.k4health.org/toolkits/kenya-health/adolescent-sexual-and-reproductive-health-and-rights. Accessed 8 Mar 2019.

[18] Essop R, Tolla TH, Lynch I, Makoae M (2018). “They tell you about the risks”:exploring sources of sexuality education among very young adolescents in rural Mpumalanga. South African J Child Heal.

[19] Igbokwe UL, Ogbonna CS, Ezegbe BN, Nnadi EM, Eseadi C (2019). Viewpoint on family life and HIV education curriculum in Nigerian secondary schools. J Int Med Res.

[20] Minet T, Eyasu H, Simon A, Afewerki W, Henok K, Russom T. Associates of Comprehensive HIV/AIDS Knowledge and Acceptance Attitude among Male Youth Aged 15–24: Comparison Study among Ivory Coast, Cameroon and Gabon. J AIDS Clin Res. 2016;7(10). Available from: https://www.omicsonline.org/open-access/associates-of-comprehensive-hivaids-knowledge-and-acceptance-attitude-among-male-youth-aged-1524-comparison-study-among-ivory-coas-2155-6113-1000618.php?aid=80140. Accessed 8 Mar 2019.

[21] Kejela G, Oljira L, Dessie Y, Misker D (2015). Comprehensive HIV/AIDS Knowledge Level among Out-of-School Youths in Wayu Tuka District, Western Ethiopia. Eur J Prev Med.

[22] Ajide KB, Balogun FM (2018). Knowledge of HIV and intention to engage in risky sexual behaviour and practices among senior school adolescents in Ibadan, Nigeria. Arch basic Appl Med.

[23] Woog V, Kågesten A. The Sexual and Reproductive Health Needs of Very Young Adolescents Aged 10–14 in Developing Countries : What Does the Evidence Show ? [Internet]. New York; 2017. Available from: https://www.guttmacher.org/sites/default/files/report_pdf/srh-needs-very-young-adolescents-report_0.pdf. Accessed 8 Mar 2019.

[24] Oginni AB, Adebajo SB, Ahonsi BA (2017). Trends and Determinants of Comprehensive Knowledge of HIV among Adolescents and Young Adults in Nigeria: 2003–2013. Afr J Reprod Heal.

[25] Dinaj-koci V, Lunn S, Deveaux L, Wang B, Chen X, Li X (2014). Adolescent age at time of receipt of one or more sexual risk reduction interventions. J Adolesc Heal.

[26] Pettifor Audrey, Stoner Marie, Pike Carey, Bekker Linda-Gail (2018). Adolescent lives matter. Current Opinion in HIV and AIDS.

[27] National Agency For the Control of AIDS. National HIV strategy for adolescents and young people: 2016–2020 [Internet]. 2016. Available from: https://www.ilo.org/wcmsp5/groups/public/%2D%2D-ed_protect/%2D%2D-protrav/%2D%2D-ilo_aids/documents/legaldocument/wcms_532857.pdf. Accessed 8 Mar 2019.

[28] Haberland N, Rogow D (2015). Sexuality education: emerging trends in evidence and practice. J Adolesc Heal.

[29] Patton GC, Ross DA, Santelli JS, Sawyer SM, Viner RM, Kleinert S (2014). Next steps for adolescent health: a lancet commission. Lancet.

[30] Sawyer SM, Afifi RA, Bearinger LH, Blakemore SJ, Dick B, Ezeh AC (2012). Adolescence: A foundation for future health. Lancet.

