# Supplementary material for: HIV comprehensive knowledge and prevalence among young adolescents in Nigeria: evidence from Akwa Ibom AIDS indicator survey, 2017
Source: BMC Public Health. 2020 Jan 13;20:45. doi: 10.1186/s12889-019-7890-y (PMC6956480; doi:10.1186/s12889-019-7890-y)
Supplement: Supplementary file 1 — Additional file 1. Akwa Ibom AIDS Indicator Survey Adolescent Individual Questionnaire (10–14 yrs). [file 12889_2019_7890_MOESM1_ESM.pdf]

AKAIS Adolescent Individual Questionnaire

Contents

CONSENT AND ELIGIBILITY CHECK 2
MODULE 1: SOCIO-DEMOGRAPHIC CHARACTERISTICS 6
MODULE 2: HIV KNOWLEDGE 9
MODULE 3: HIV PREVENTION INVERVENTIONS 11
MODULE 4: SEXUAL BEHAVIOR 12
MODULE 5: HIV RISK PERCEPTION 16
MODULE 6: SOCIAL NORMS, INTENTION TO ABSTAIN, SELF-EFFICACY AND ASSERTIVENESS 17
MODULE 7: HIV TESTING 18
MODULE 8: HIV STIGMA 19
MODULE 9: ALCOHOL AND DRUGS 20
MODULE 10: PARENTAL SUPPORT 21

CONSENT AND ELIGIBILITY CHECK

NO.	VARNAME	QUESTIONS	CODING CATEGORIES	SKIPS/FILTERS	CORE/SUPPLEMENT	AKAIS ONLY	NOTES
1		FIRST NAME OF THE PARTICIPANT FROM HOUSEHOLD ROSTER<br><br>CONFIRM NAME WITH PARTICIPANT	FIRST NAME:________________________				
2		AGE OF PARTICIPANT FROM HOUSEHOLD ROSTER<br><br>ASK PARTICIPANT: “How old were you at your last birthday?”<br><br>Does (NAME) confirm their age at last birthday?<br><br>*IF AGE DIFFERENT FROM HOUSEHOLD ROSTER, PLEASE VERIFY CORRECT AGE OF PARTICIPANT*	AGE:_______________________	MUST BE 10-14 YRS			
3		SCAN BARCODE OF PARTICIPANT					

4		RECORD SEX OF THE RESPONDENT	MALE = 1<br>FEMALE = 2		C		
5		TO INTERVEIWER: FOLLOW THE NEXT STEPS<br>1) CONSENT (NAME)'S PARENT/GUARDAIN<br>2) ASSESS (NAME)'S ELIGIBILITY (LANGUAGE, HEARING ABILITY, COGNITIVE ABILITY)<br>3) GAIN ASSENT FROM (NAME)					
6		FROM PARENTAL PERMISSION FORM:<br>TO THE PARENT/GUARDIAN<br><br>Did you give permission for (NAME) to take part in this study?	YES = 1<br>NO = 2	IF YES → 8			
7		FOR PARENT/GUARDIAN:<br>What are the reasons that you do not want (NAME) to participate in the survey?<br><br>DON'T READ OUT LOUD<br><br>PROBE<br><br>SELECT ALL THAT APPLY	NO TIME= 1<br>NOT COMFORTABLE WITH INTERVIEW= 2<br>DOESN'T LIKE QUESTIONS ON SEX= 3<br>DOESN'T LIKE BLOOD DRAW= 4<br>DOESN'T WANT TO GET RESULTS= 5<br>WORRIES ABOUT CONFIDENTIALITY= 6<br>DOESN'T WANT TO BE TESTED FOR HIV=7<br>ALREADY KNOWS HIV POSITIVE=8<br>OTHER (SPECIFY)=96<br>______________________________<br>DON'T KNOW=98<br>REFUSES TO SAY= 99	END INTERVIEW			

8		FOR THE INTERVIEWER, DON'T READ OUT LOUD<br><br>Is this (NAME) eligible for the survey based on assessment (Step 2)?	YES = 1<br>NO = 2	IF YES →10			
9		Reason for ineligibility:	HEARING DISABILITY=1<br>DOES NOT SPEAK A LANGUAGE THE SURVEY TEAM CAN ACCOMMODATE=2<br>VISUAL IMPAIRMENT=3<br>COGNITIVE DISABILITY=4<br>OTHER=98 (SPECIFY)	END INTERVIEW			
10		FROM ASSENT FORM:TO PARTICIPANT:<br><br>Did you agree to take part in this study?	YES = 1<br>NO = 2	IF YES→12			
11		FOR PARTICIPANT:<br><br>What are the reason that you do not want to participant in the survey?	NO TIME= 1<br>NOT COMFORTABLE WITH INTERVIEW= 2<br>DOESN'T LIKE QUESTIONS ON SEX= 3<br>DOESN'T LIKE BLOOD DRAW= 4<br>DOESN'T WANT TO GET RESULTS OF HIV TEST= 5<br>WORRIES ABOUT CONFIDENTIALITY= 6<br>DOESN'T WANT TO BE TESTED FOR HIV=7<br>ALREADY KNOWS HIV POSITIVE=8<br>OTHER (SPECIFY)=96<br>________________________________<br>DON'T KNOW=98<br>REFUSES TO SAY= 99	END INTERVIEW			

12		What Language do you prefer for our discussion today?	ENGLISH=1<br>ANNANG=2<br>IBIBIO=3<br>ORO=4<br>PIDGIN=5				

MODULE 1: SOCIO-DEMOGRAPHIC CHARACTERISTICS

NO.	VARNAME	QUESTIONS	CODING CATEGORIES	SKIPS/FILTERS
101		Do you go currently to school?	YES = 1<br>NO = 2<br>DON'T KNOW = 998<br>REFUSED TO SAY = 999	IF NO, DK, REFUSED → 105
102		During the last school week, did you miss any school days for any reason?	YES = 1<br>NO = 2<br>DON'T KNOW = 998<br>REFUSED TO SAY = 999	IF NO, DK, REFUSED → 104
103		Why did you miss school?<br><br>SELECT ALL THAT APPLY	I WAS SICK = 1<br>I DIDN'T FEEL SAFE GOING TO/WHILE IN SCHOOL = 2<br>MY PARENTS DIDN'T TAKE ME TO SCHOOL=3<br>I DON'T LIKE SCHOOL = 4<br>I HAD TO LOOK AFTER MY FAMILY= 5<br>THERE WAS NOT ENOUGH MONEY TO SEND ME TO SCHOOL = 6<br>SCHOOL IS TOO FAR AWAY = 7<br>I HAD TO WORK = 8<br>I HAD A CHILD OR I AM PREGNANT (GIRLS ONLY) = 9<br>I MISSED SCHOOL BECAUSE OF MY PERIOD (MENSTRUATION) (GIRLS ONLY) = 10<br>OTHER (SPECIFY) = 96<br>______________________________	

NO.	VARNAME	QUESTIONS	CODING CATEGORIES	SKIPS/FILTERS
			DON’T KNOW = 998<br>REFUSED TO SAY = 999	
104		What level of school are you currently in?	IN PRIMARY = 1<br>COMPLETED PRIMARY, NOT IN JSS = 2<br>COMPLETED PRIMARY, IN JSS = 3<br>COMPLETED JSS = 4<br>IN SSS = 5<br>COMPLETED SSS =6<br>QUARANIC SCHOOL=7<br>OTHER (SPECIFY)………………… = 8<br>DON’T KNOW = 98<br>REFUSED TO SAY = 99	GO TO 201 (NEXT MODULE)
105		When was the last time you regularly attended school? Would you say it was less than a year ago or more than a year ago?	LESS THAN 1 YEAR = 1<br>1 YEAR OR LONGER = 2<br>DON’T KNOW = 998<br>REFUSED TO SAY = 999	
106		Have you ever attended school?	YES = 1<br>NO = 2<br>DON’T KNOW = 998<br>REFUSED TO SAY = 999	IF YES→108

NO.	VARNAME	QUESTIONS	CODING CATEGORIES	SKIPS/FILTERS
107		What is the **main** reason you never attended school?<br><br>SELECT ONLY ONE RESPONSE	I HAVE BEEN SICK = 1<br>I DONDT FEEL SAFE GOING TO/WHILE IN SCHOOL = 2<br>MY PARENTS DON'T TAKE ME TO SCHOOL = 3<br>I DON'T LIKE SCHOOL = 4<br>I HAVE TO LOOK AFTER MY FAMILY = 5<br>THERE'S NOT ENOUGH MONEY TO SEND ME TO SCHOOL = 6<br>SCHOOL IS TOO FAR AWAY = 7<br>I HAVE TO WORK = 8<br>I HAVE A CHILD OR I AM PREGNANT (GIRLS ONLY) = 9<br>OTHER (SPECIFY) = 96<br>______________________________<br>DON'T KNOW = 998<br>REFUSED TO SAY = 999	END MODULE
108		What is the highest level of school that you have completed?	NURSERY/KINDERGARTEN = 1<br>SOME PRIMARY = 2<br>COMPLETED PRIMARY, NOT IN JSS = 3<br>COMPLETED PRIMARY, IN JSS = 4<br>SOME JSS=5<br>COMPLETED JSS = 6<br>SOME SSS = 7<br>COMPLETED SSS =8<br>QUARANIC SCHOOL=9<br>ISLAMIYYA=10<br>OTHER (SPECIFY) = 96<br>______________________________<br>DON'T KNOW = 998	

NO.	VARNAME	QUESTIONS	CODING CATEGORIES	SKIPS/FILTERS
			REFUSED TO SAY = 999	

MODULE 2: HIV KNOWLEDGE

READ: Now I would like to ask you some questions about what you know about some things related to health.

NO.	VARNAME	QUESTIONS	CODING CATEGORIES	SKIPS/FILTERS
201		Have you heard of a disease called HIV/AIDS?	YES = 1<br>NO = 2<br>DON'T KNOW = 998<br>REFUSED TO SAY = 999	IF NO, DK,<br>REFUSED → 302
202		How did you hear about HIV/AIDS?<br><br>SELECT ALL THAT APPLY	SCHOOLS/TEACHERS = 1<br>PARENTS/GUARDIAN = 2<br>OTHER FAMILY MEMBERS = 3<br>FRIENDS/PEERS = 4<br>RELIGIOUS LEADERS = 5<br>INTERNET = 6<br>MOBILE PHONE = 7<br>HOSPITAL/CLINIC = 8<br>TELEVISION/FILM = 9	

			RADIO = 10<br>HEALTH WORKERS = 11<br>OTHER (SPECIFY) = 96<br>_______________________________<br>DON'T KNOW = 998<br>REFUSED TO SAY = 999	
203		Have you <u>ever</u> discussed HIV or AIDS with your parents or guardian?	YES = 1<br>NO = 2<br>DON'T KNOW = 998<br>REFUSED TO SAY = 999	
204		Can a healthy looking person have HIV?	YES = 1<br>NO = 2<br>DON'T KNOW = 998<br>REFUSED TO SAY = 999	
205		How can HIV be transmitted?<br><br>SELECT ALL THAT APPLY	UNPROTECTED SEX WITH AN INFECTED PERSON = 1<br>SHARING SHARP OBJECTS = 2<br>CONTACT WITH BLOOD = 3<br>BLOOD TRANSFUSION = 4<br>MOTHER TO CHILD = 5<br>OTHER (SPECIFY) = 96<br>_______________________________<br>DON'T KNOW = 998<br>REFUSED TO SAY = 999	
206		Are there medicines that people with HIV can take to help them live longer?	YES = 1	

			NO = 2<br>DON'T KNOW = 998<br>REFUSED TO SAY = 999	

MODULE 3: HIV PREVENTION INVERVENTIONS

NO.	VARNAME	QUESTIONS	CODING CATEGORIES	SKIPS/FILTERS
301		How can someone protect himself/herself from HIV?<br><br>DON'T READ OPTIONS<br><br>SELECT ALL THAT APPLY	NOT HAVING SEX = 1<br>USING CONDOM = 2<br>NOT SHARING SHARP OBJECTS = 3<br>NOT HAVING CONTACT WITH BLOOD = 4<br>OTHER (SPECIFY) = 96<br>______________________________<br>DON'T KNOW = 998<br>REFUSED TO SAY = 999	
302		Have you ever heard of condoms?	YES = 1<br>NO = 2<br>REFUSED TO SAY = 999	IF NO, REFUSED TO SAY → 401 (NEXT MODULE)
303		What is it used for?<br><br>SELECT ALL THAT APPLY	PREVENTION OF PREGNANCY =1<br>PREVENTION OF HIV = 2<br>PREVENTION OF OTHER INFECTIONS THAT CAN BE TRANSMITTED THROUGH SEX = 3<br>OTHER (SPECIFY) = 96	

NO.	VARNAME	QUESTIONS	CODING CATEGORIES	SKIPS/FILTERS
			___________________________<br>DON'T KNOW = 998<br>REFUSED TO SAY = 999	

MODULE 4: SEXUAL BEHAVIOR

QUESTIONS 401-703 (MODULES 4-7) ARE ONLY FOR CHILDREN AGED 12-14 YEARS. CHECK AGE OF CHILD (QUESTION 2). IF CHILD IS 10-11 YEARS OLD, GO TO QUESTION 801 (MODULE 8).

READ: The next questions ask about sexual behavior. There are no right or wrong answers. Your responses will not be linked to you in any way or shared with anyone, including your parents.

PLEASE LOOK OUT FOR SIGNS OF DISTRESS IN CHILD WHEN ASKING THE FOLLOWING SEXUAL BEHAVIOR QUESTIONS. IF THE CHILD SEEMS DISTRESSED, ASK CHILD WHETHER HE/SHE WANTS TO CONTINUE. IF THE CHILD IS IN DISTRESS, DISCONTINUE AND MOVE TO THE NEXT MODULE.

TO MOVE TO THE NEXT SECTION, TOGGLE FROM THE RIGHT TOP CORNER OF THE TABLET, AND SCROLL DOWN TO THE NEXT MODULE.

NO.	VARNAME	QUESTIONS	CODING CATEGORIES	SKIPS/FILTERS
401		Have you ever been married?	YES = 1<br>NO = 2<br>REFUSED TO SAY = 999	IF NO→403
402		At what age were you first married?	AGE AT MARRIAGE (IN YEARS): ___ ___<br>DON'T KNOW = 998<br>REFUSED TO SAY = 999	→406

NO.	VARNAME	QUESTIONS	CODING CATEGORIES	SKIPS/FILTERS
403		Have you ever heard of sex?	YES = 1<br>NO = 2<br>DON'T KNOW = 998<br>REFUSED TO SAY = 999	SKIP IF MARRIED<br><br>IF NO, DK, REFUSED → NEXT MODULE
404		Have you ever had sex?<br><br>By sex, we mean any sexual contact, that is, vaginal, oral or anal sex.	YES = 1<br>NO = 2<br>DON'T KNOW = 998<br>REFUSED TO SAY = 999	IF NO, DK, REFUSED → NEXT MODULE
405		How did you do it?<br><br>DON'T READ OPTIONS<br><br>SELECT ALL THAT APPLY	VAGINAL SEX = 1<br>ORAL SEX = 2<br>ANAL SEX = 3<br>REFUSED TO SAY = 999	
406		How old were you when you had sex for the first time?	AGE IN YEARS: __ __<br><br>DON'T KNOW = 998<br>REFUSED TO SAY = 999	
407		What was the **main** reason that you had sex for the first time?<br><br>DO NOT READ OUT LOUD	IT JUST HAPPENED = 1<br>MY FRIENDS PRESSURED ME TO HAVE SEX = 2<br>TO PROVE MY LOVE/TO FEEL LOVED = 3<br>I WANTED TO HAVE SEX/CURIOUS = 4	

NO.	VARNAME	QUESTIONS	CODING CATEGORIES	SKIPS/FILTERS
		SELECTED ONLY ONE RESPONSE	MY BOYFRIEND/GIRLFRIEND WANTED TO HAVE SEX = 5<br>I WAS TRICKED, THREATENED OR FORCED = 6<br>FOR MONEY/GIFTS = 7<br>I WANTED TO HAVE A BABY = 8<br>OTHER (SPECIFY) = 96<br>______________________________<br>DON'T KNOW = 998<br>REFUSED TO SAY = 999	
408		How old was the first person you had sex with? Were they older, younger or same age as you?	OLDER THAN ME = 1<br>SAME AGE AS ME = 2<br>YOUNGER THAN ME = 3<br>DON'T KNOW = 998<br>REFUSED TO SAY = 999	
409		Did you use a condom the <u>first</u> time you had sex?	YES = 1<br>NO = 2<br>DON'T KNOW = 998<br>REFUSED TO SAY = 999	IF NO, REFUSED TO SAY (FROM 409) OR NEVER HEARD OF CONDOM (FROM 302) → 412
410		The <u>last</u> time you had sex, was a condom used?	YES = 1<br>NO = 2<br>DON'T KNOW = 998	

NO.	VARNAME	QUESTIONS	CODING CATEGORIES	SKIPS/FILTERS
			REFUSED TO SAY = 999	
411		How often do you use a condom during sex: Always, Sometimes, Never?<br>By sex, we mean vaginal, oral or anal sex.	ALWAYS = 1<br>SOMETIMES = 2<br>NEVER = 3<br>DON'T REMEMBER = 4<br>DON'T KNOW = 998<br>REFUSED TO SAY = 999	
412		How many different people have you ever had sex with?<br><br>CAN NOT BE ZERO	NUMBER OF PARTNERS: ___<br>DON'T KNOW = 998<br>REFUSED TO SAY = 999	
413		Sometimes people have sex to get material support. Material support means helping you to pay for things or giving you gifts or money.<br><br>Have you ever had sex with someone for material support?	YES = 1<br>NO = 2<br>DON'T KNOW = 998<br>REFUSED TO SAY TO SAY = 999	
414		Have you ever been pregnant?<br><br>GIRLS ONLY	YES = 1<br>NO = 2<br>DON'T KNOW = 998<br>REFUSED TO SAY = 999	

NO.	VARNAME	QUESTIONS	CODING CATEGORIES	SKIPS/FILTERS
415		Have you <u>ever</u> talked with a parent or guardian about sex?	YES = 1<br>NO = 2<br>DON'T KNOW = 998<br>REFUSED TO SAY = 999	

MODULE 5: HIV RISK PERCEPTION

IF RESPONDENT HAS NOT HEARD OF HIV, DON'T KNOW, OR REFUSED (QUESTION 201), GO TO QUESTION 601 (NEXT MODULE).

NO.	VARNAME	QUESTIONS	CODING CATEGORIES	SKIPS/FILTERS
501		How likely do you think is it that you can get HIV: Very Likely, Somewhat Likely, or Not Likely?	VERY LIKELY = 1<br>SOMEWHAT LIKELY = 2<br>NOT LIKELY = 3<br>I ALREADY HAVE HIV = 4<br>DON'T KNOW = 998<br>REFUSED TO SAY = 999	IF NOT LIKELY → 503<br><br>IF HAVE HIV, DK, REFUSED → 601 (NEXT MODULE)
502		What is the <u>main</u> reason you think you are likely to get HIV?<br><br>DON'T READ ALOUD	I HAVE HAD SEX WITHOUT A CONDOM = 1<br>I HAVE OR HAD MANY BOYFRIENDS/GIRLFRIENDS = 2<br>I HAVE HAD BLOOD TRANSFUSIONS = 3<br>MY MOTHER/FATHER HAS HIV = 4<br>I DON'T TRUST MY BOYFRIEND/GIRLFRIEND = 5<br>I AM SICK = 6<br>MY BOYFRIEND/GIRLFRIEND IS SICK OR HAS DIED = 7	ALL → 601 (NEXT MODULE)

		PROBE FOR MAIN REASON	OTHER (SPECIFY) = 96<br>______________________________<br>DON'T KNOW = 998<br>REFUSED TO SAY = 999	
503		What is the <u>main</u> reason you think you are not likely to get HIV?<br><br>DON'T READ ANSWERS ALOUD<br><br>PROBE FOR MAIN REASON	I DO NOT HAVE SEX = 1<br>I USE CONDOMS = 2<br>I HAVE ONLY ONE PARTNER = 3<br>I TRUST MY PARTNER = 4<br>OTHER (SPECIFY) = 96<br>______________________________<br>DON'T KNOW = 998<br>REFUSED TO SAY = 999	

MODULE 6: SOCIAL NORMS, INTENTION TO ABSTAIN, SELF-EFFICACY AND ASSERTIVENESS

IF RESPONDENT HAS NOT HEARD OF SEX, DON'T KNOW, OR REFUSED (QUESTION 401), GO TO QUESTION 701 (NEXT MODULE).

NO.	VARNAME	QUESTIONS	CODING CATEGORIES	SKIPS/FILTERS
601		How many of your friends do you think are having sex?	ALL = 1<br>MOST = 2<br>SOME = 3<br>A FEW = 4<br>NONE = 5<br>DON'T KNOW = 998<br>REFUSED TO SAY = 999	SKIP IF MARRIED (from 401)

602		Are you pressured by your friends or boyfriend/girlfriend to have sex?	YES = 1<br>NO = 2<br>DON'T KNOW = 998<br>REFUSED TO SAY = 999	
603		If you did not want to have sex with someone, could you tell them that you do not want to have sex with them?	YES = 1<br>NO = 2<br>DON'T KNOW = 998<br>REFUSED TO SAY = 999	

MODULE 7: HIV TESTING

IF RESPONDENT HAS NOT HEARD OF HIV, DON'T KNOW, OR REFUSED (QUESTION 201), GO TO QUESTION 801 (NEXT MODULE).

READ: I would now like to ask you some questions about HIV testing.

NO.	VARNAME	QUESTIONS	CODING CATEGORIES	SKIPS/FILTERS
701		Have you ever been tested for HIV?	YES = 1<br>NO = 2<br>DON'T KNOW = 998<br>REFUSED TO SAY = 999	IF NO, DK, REFUSED → 801 (NEXT MODULE)
702		Did you receive the results of any of your HIV tests?	YES = 1<br>NO = 2	

			DON'T KNOW = 998<br>REFUSED TO SAY = 999	IF NO, DK, REFUSED → 801 (NEXT MODULE)
703		What was the result of that HIV test?	HIV POSITIVE = 1<br>HIV NEGATIVE = 2<br>INDETERMINATE = 3<br>DON'T KNOW = 998<br>REFUSED TO SAY = 999	

MODULE 8: HIV STIGMA

IF RESPONDENT HAS NOT HEARD OF HIV, DON'T KNOW, OR REFUSED (QUESTION 201), GO TO QUESTION 901 (NEXT MODULE).

READ: I would now like to ask you some questions about how you feel about people with HIV.

	VARNAME	QUESTIONS	CODING CATEGORIES	SKIPS/FILTERS
801		Would you be willing to share food with someone who has HIV?	YES = 1<br>NO = 2<br>DON'T KNOW = 998<br>REFUSED TO SAY = 999	

802		Would you play with someone who has HIV?	YES = 1<br>NO = 2<br>DON'T KNOW = 998<br>REFUSED TO SAY = 999	

MODULE 9: ALCOHOL AND DRUGS

READ: I would like to ask you some questions about alcohol and drugs or substances that you may have taken that were not given to you by a doctor. Your answers will not be told to anyone, not even your parents.

NO.	VARNAME	QUESTIONS	CODING CATEGORIES	SKIPS/FILTERS
901		Have you ever taken alcohol?	YES = 1<br>NO = 2<br>DON'T KNOW = 998<br>REFUSED TO SAY = 999	IF NO, DK, REFUSED<br>→ 903
902		During the past 1 month, on how many days did you have at least one drink containing alcohol?	NUMBER OF DAYS: ___ ___<br>DON'T KNOW = 998<br>REFUSED TO SAY = 999	MAX = 31

903		Have you ever tried any substances to make you high?	YES = 1<br>NO = 2<br>DON'T KNOW = 998<br>REFUSED TO SAY = 999	IF NO, DK, REFUSED → 1001 (NEXT MODULE)
904		What substances/drugs have you ever tried to make you high?<br><br>DO NOT READ RESPONSES, PROBE FOR MULTIPLE RESPONSES	COCAINE = 1<br>HEROINE = 2<br>INDIAN HEMP = 3<br>TRANQUILIZERS = 4<br>CODEINE = 5<br>SNIFFING PETROL = 6<br>SNIFFING TOBACCO = 7<br>SNIFFING BURNING RUBBER = 8<br>SNIFING GUM = 9<br>INHALING SEWAGE/GUTTERS = 10<br>OTHER (SPECIFY) = 96<br>______________________________<br>DON'T KNOW = 998<br>REFUSED TO SAY = 999	

MODULE 10: PARENTAL SUPPORT

READ: Finally, I would like to ask you some questions about your relationship with your parents/guardian.

NO.	VARNAME	QUESTIONS	CODING CATEGORIES	SKIPS/FILTERS
1001		Do your parents/guardians understand your problems and worries?	ALWAYS = 1<br>MOST OF THE TIME = 2<br>SOMETIMES = 3<br>RARELY = 4<br>NEVER = 5<br>DON'T KNOW = 998<br>REFUSED = 999	
1002		Do your parents/guardians really know what you are doing with your free time when you are not at school or work?	ALWAYS = 1<br>MOST OF THE TIME = 2<br>SOMETIMES = 3<br>RARELY = 4<br>NEVER = 5<br>DON'T KNOW = 998<br>REFUSED = 999	
